# Cyclooxygenase-2 and B-cell lymphoma-2 expression in cystitis glandularis and primary vesicle adenocarcinoma

**DOI:** 10.1186/1471-2490-14-2

**Published:** 2014-01-03

**Authors:** Zhongxing Li, Guangcheng Ge, Rui Feng, Dan Wu, Bin Shen, Xing Wang, Yan Cui, Junrong Li, Xiaobing Ju

**Affiliations:** 1 Department of Urology, The No.2 People’s Hospital of Zhenjiang, affiliated with Jiangsu University, Zhenjiang, China; 2 Department of Epidemiology, Jiangsu University, Zhenjiang, China; 3 Department of Urology, The First Affiliated Hospital of Nanjing Medical University, 300 Guangzhou Road, Nanjing 210029, China

**Keywords:** Cyclooxygenase-2, Bcl-2, Cystitis

## Abstract

**Background:**

Although cystitis glandularis (CG) is a common benign urinary bladder epithelial abnormality, it remains unclear whether CG is a premalignant lesion. Cyclooxygenase-2 (COX-2) and B-cell lymphoma-2 (Bcl-2) overexpression has recently been reported as a potential tumor initiator or promoter. We evaluated and compared COX-2 and Bcl-2 expression in CG, chronic cystitis (CC), and primary vesicle adenocarcinoma (ADC) tissues.

**Methods:**

We conducted a retrospective study to investigate COX-2 and Bcl-2 levels in CG and ADC. We obtained tissue samples from 75 patients (including 11 cases of CC, 30 typical cases of CG (CGTP), 30 cases of intestinal CG (CGIT), and 4 cases of ADC) between 1989 and 2009 from the Surgical Pathology Archives of the No. 2 People’s Hospital of Zhenjiang, affiliated with Jiangsu University. COX-2 and Bcl-2 immunohistochemical staining was performed on all tissues. Nine normal bladder epithelial specimens were evaluated as control samples. Correlations between COX-2 and Bcl-2 expression in CG were also analyzed.

**Results:**

COX-2 and Bcl-2 expression was higher in the ADC group compared to other groups (p < 0.05). COX-2 and Bcl-2 levels were higher in the CGIT group compared to the CGTP group (p = 0.000 for both). The CGIT and CGTP groups both showed higher COX-2 expression compared to the CC group (p = 0.000 for both). There was no difference in Bcl-2 expression between the CGTP and CC groups (p = 0.452). Additionally, the difference in COX-2 and Bcl-2 expression between the control and CC groups was also insignificant (p = 0.668 and p = 0.097, respectively). Finally, we found that COX-2 and Bcl-2 levels were positively related (*r* = 0.648, p = 0.000).

**Conclusion:**

COX-2 and Bcl-2 overexpression in the CG group suggests that CG, particularly the intestinal type, may be a premalignant lesion that converts into a tumor in the presence of carcinogens.

## Background

Cystitis glandularis (CG) is a common benign epithelial abnormality that occurs in the presence of chronic inflammation [1,2]. Based on morphology and behavior, CG has been subdivided into two subtypes. Typical CG (CGTP) is characterized by nests of columnar epithelial cells within the bladder lamina propria that form glandular structures. The intestinal type (CGIT) has similar glandular architecture in the lamina propria but contains abundant mucin-secreting goblet cells in the lining epithelium [3]. Although the cause of CG is debatable [4], it is generally agreed that in the presence of chronic inflammation, the bladder mucosa becomes hyperproliferative. When proliferation projects into the lamina propria, epithelial nests (von Brunn’s nests) [5] and cystitis cystica or glandular lesions (CG) form [5,6]. CG, particularly the intestinal type, has been described as premalignant; however, not all studies agree with this conclusion [3,7]. Due to rare reported instances of CG progression to adenocarcinoma or CG associated with adenocarcinoma, the relationship between CG and subsequent bladder adenocarcinoma remains unclear.

Cyclooxygenase is an important enzyme that catalyzes the conversion of arachidonic acid to prostaglandin. COX-1 is constitutively expressed in most tissues and regulates multiple physiological processes. In contrast, COX-2 is frequently undetectable in normal tissues, but can be induced by a variety of stimuli, including mitogens, cytokines, growth factors, and hormones, thereby resulting in inflammation and cellular proliferation [8]. COX-2 overexpression is observed in chronic inflammation as well as in various tumors, including bladder, prostate, colon, and lung [9-12]. To this end, we assessed the differential expression of COX-2 in normal bladder transitional cell tissue, chronic cystitis, two subtypes of CG, and bladder adenocarcinoma tissue. In addition, we determined if COX-2 expression is associated with expression of Bcl-2, a regulator and marker of apoptosis.

## Methods

### Patient samples

Tissues from 75 patients, including 60 cases of CG, 11 cases of chronic cystitis (CC), and 4 cases of primary vesicle adenocarcinoma (ADC), were obtained from the Surgical Pathology Archives of the No. 2 People’s Hospital of Zhenjiang, affiliated with Jiangsu University between 1989 and 2009. Normal bladder specimens from nine subjects who underwent cystectomy for benign causes were used as controls. The Institutional Review Board of Nanjing Medical University (Nanjing, China) approved this study. At the time of patient recruitment, written informed consent was obtained from all participants. We classified CG into CGTP and CGIT based on routine hematoxylin and eosin-stained sections. One of the ADC patients had the intestinal type of CG and interrupted use of antibiotics rather than intravesical instillation of the anticancer agent. Another patient had neurogenic bladder with suprapubic cystostomy for fifteen years. The other two patients had classic bladder exstrophy with an unsuccessful initial closure.

### Immunohistochemistry and staining evaluation

Sections (5 μm thick) were cut from formalin-fixed, paraffin-embedded tissue blocks and stained with hematoxylin and eosin. Additional sections from appropriately selected blocks were cut for use in immunohistochemical analyses as described previously [13,14]. Two primary antibodies were used for immunochemical staining: monoclonal antibodies against COX-2 (monoclonal mouse anti-human D12; Santa Cruz Biotechnology, USA) and Bcl-2 (monoclonal mouse anti-human; Dako, Carpinteria, USA). Briefly, sections were baked for 2 hour at 72°C and deparaffinized by sequential immersion in xylene, 95% ethanol, 80% ethanol, and distilled water for 5 min each. Next, slides were placed in an autoclave containing antigen retrieval solution (0.1 M citrate buffer from BDH at pH 6.0) for 2 min at 121°C. Diluted primary antibodies (100 μl) were applied to the sections and slides were incubated in a humid chamber for 2 h at 37°C. Slides were rinsed gently with PBS and placed in a fresh PBS bath for 5 min. Next, one or two drops of diluted biotinylated secondary goat anti-mouse antibodies (Dako Cytomation) were applied to the sections and the slides were incubated in a humid chamber for 2 h at 37°C. After rinsing, one or two drops of streptavidin-horseradish peroxidase reagent (Dako Cytomation) was added to the sections and slides were incubated for 30 min at 37°C. Next, the prepared DAB substrate chromogen solution was applied to the sections and slides were incubated in the dark at room temperature for 5 min. Mayer's hematoxylin stain was used as a counterstain, and slides were dehydrated and mounted.

Staining was evaluated as described previously [14,15]. Briefly, two pathologists who were unaware of the clinical data scored immunohistochemical expression in a semi-quantitative fashion. Expression levels were assessed by evaluating the percentage of the cell that was stained, and recorded as absent, weakly, moderately, or markedly positive. (5-25% indicated weakly, 25-50% indicated moderately, >50% indicated markedly) Using light microscopy, the mean percentage of \ positively stained cells in each section was calculated from three dense, medium, and light staining areas. In each area, the percentage of brown stained cells was calculated from the total number of countable cells in five high power fields. Therefore, expression scoring was determined to be discernible and reproducible.

### Statistical analyses

Kruskal-Wallis H tests were employed to evaluate differences in the amount of COX-2 and Bcl-2 expression among control, CC, CGTP, CGIT and ADC specimens. To further compare the expression of two groups, we performed Mann–Whitney U tests. Spearman’s tests were used to analyze the correlation between COX-2 and Bcl-2 expression in CG specimens. P values less than 0.05 were considered significant, and all P values are two-sided. All analyses were performed using SPSS version 13.0 (SPSS, USA).

## Results

**Figure 1:**
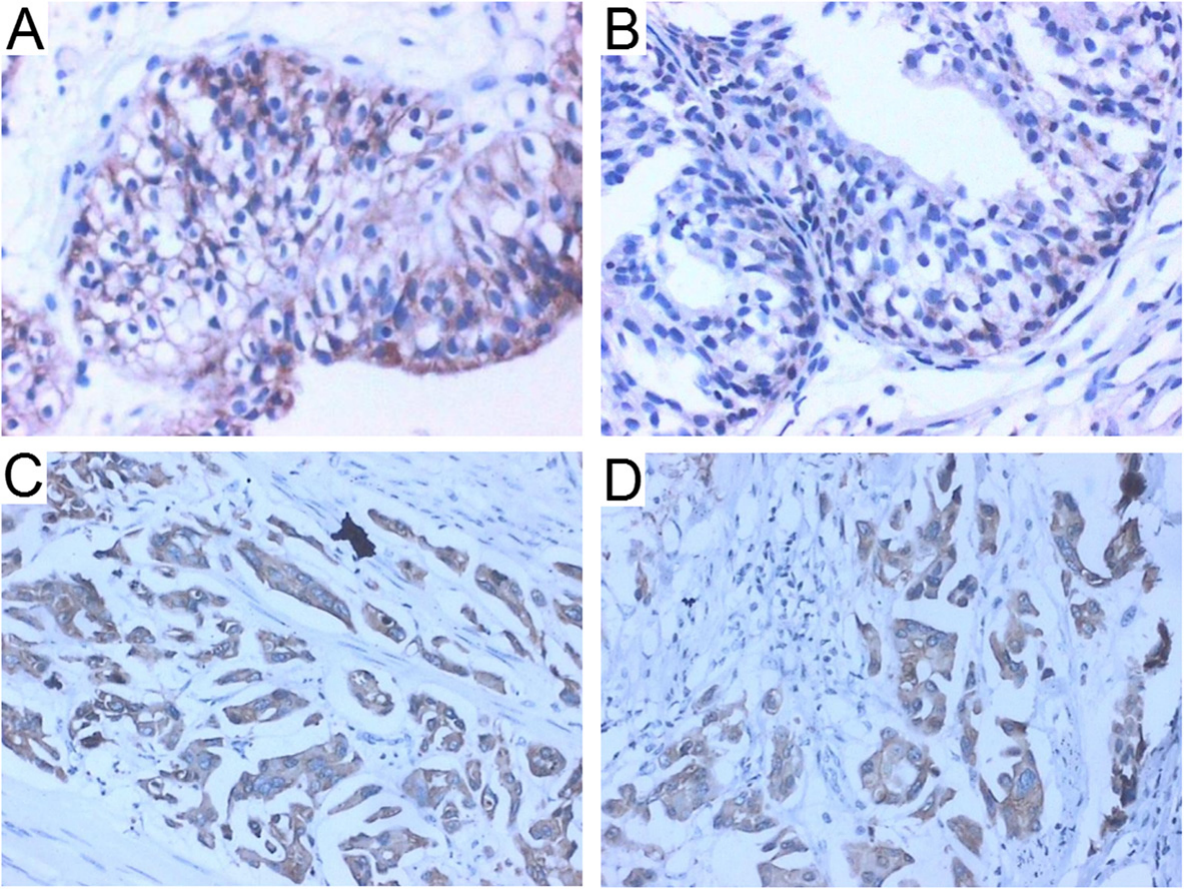
**Immunohistochemical staining to evaluate Cox-2 and Bcl-2 localization (original magnification 400×). A**. Cox-2 expression in CGIT. **B**. Bcl-2 expression in CGIT. **C**. COX-2 expression in **ADC. D**. Bcl-2 expression in **ADC**.

The immunohistochemical staining results are summarized in Table 1, and typical examples from the CGIT and ADC groups are shown in Figure 1. There were significant differences in COX-2 and Bcl-2 expression among the five groups (χ^2^ = 58.917, p = 0.000; χ^2^ = 50.993, p = 0.000, respectively). The ADC group showed the highest levels of COX-2 and Bcl-2 expression compared to the other groups (p < 0.05). COX-2 and Bcl-2 expression levels were higher in the CGIT group compared to the CGTP group (Z = -4.473, p = 0.000; Z = -5.580, p = 0.000, respectively), and both of these groups showed higher COX-2 expression compared to the CC group (Z = -5.227, p = 0.000; Z = -4.482, p = 0.000, respectively). However, the difference in Bcl-2 expression between the CGTP and CC groups was not significant (Z = -0.752, p = 0.452). COX-2 and Bcl-2 levels were not different between the control and CC groups (Z = -0.429, p = 0.668; Z = -1.658, p = 0.097, respectively).

**Table 1:** Immunohistochemical staining levels in control, CC, CGTP, CGIT, ADC groups

**Levels of expression**	**COX-2**					**Bcl-2**				
	**Control**	**CC**	**CGTP**	**CGIT**	**ADC**	**Control**	**CC**	**CGTP**	**CGIT**	**ADC**
Absent	8 (88.9%)	9 (81.8%)	1 (3.3%)	0 (0%)	0 (0%)	8 (88.9%)	6 (54.5%)	11 (36.7%)	0 (0%)	0 (0%)
Weak	1 (11.1%)	2 (18.2%)	18 (60.0%)	3 (10.0%)	0 (0%)	1 (11.1%)	4 (36.4%)	18 (60.0%)	9 (30.0%)	0 (0%)
Moderate	0 (0%)	0 (0%)	11 (36.7%)	22 (73.3%)	1 (25.0%)	0 (0%)	1 (9.1%)	1 (3.3%)	20 (66.7%)	2 (50.0%)
Marked	0 (0%)	0 (0%)	0 (0%)	5 (16.7%)	3 (75.0%)	0 (0%)	0 (0%)	0 (0%)	1 (3.3%)	2 (50.0%)

CC: Chronic cystitis; CGTP: Typical type of cystitis glandularis; CGIT: Intestinal type of cystitis glandularis; ADC: Adenocarcinoma of bladder.

To determine whether increased COX-2 expression was associated with up-regulation of the anti-apoptotic protein Bcl-2 in CG patients, Spearman’s tests were performed to analyze the correlation between expression of the two proteins in specimens. We found that COX-2 and Bcl-2 expression were positively related (*r* = 0.648, p = 0.000).

## Discussion

COX-2 overexpression contributes to tumorigenesis through multiple and complex mechanisms [9]. Liu et al. reported that strong COX-2 expression in murine mammary gland epithelial cells resulted in breast tumor development [16]. Nevertheless, other mouse models of skin carcinogenesis found that COX-2 plays a role in tumor promotion rather than initiation [17,18]. In the current study, we observed that COX-2 expression in CGIT and CGTP specimens were significantly higher compared to CC and control. CGIT tissue had a stronger COX-2 expression compared to CGTP. Additionally, COX-2 was aberrantly expressed in ADC tissue. These data suggest that the COX-2 overexpression in these two CG subtypes likely contribute to sensitizing premalignant lesions to genotoxic carcinogens.

Apoptosis is a programmed cell death process that depends on a balance of pro- and anti-apoptotic factors. It is vital for tissue homeostasis and defense against pathogens. Decreased apoptosis has been observed in premalignant lesions. It is well known that COX-2 overexpression increases expression of the proto-oncogene Bcl-2 and inhibits apoptosis [19]. Bcl-2, the first apoptotic regulator identified, was originally discovered as the defining oncogene in follicular lymphomas [20]. Unlike other oncogenes that increased cell proliferation, Bcl-2 inhibited programmed cell death and affected the apoptotic pathway, which are critical for cancer development [19]. Our results demonstrate that Bcl-2 expression in CGIT, but not in CGTP, was significantly higher compared to CC and control. However, ADC cases had the highest levels of Bcl-2. Additionally,, Bcl-2 expression in CG cases was positively related to COX-2 expression, similar to the report by Tsujii et al. [21]. These data suggest that impaired apoptosis may occur both in both CG subtypes and play a critical role in premalignant lesions.

Several reports have shown that adenocarcinoma of the bladder is associated with CG [22-25]. However, after more than ten years of data tracking, Corica et al. reported that none of the 53 patients with CG developed bladder cancer [26]. As a result, the association between CG and adenocarcinoma remains unclear. Although inflammation is regarded as a possible initiator of cancer [27,28] and COX-2 and Bcl-2 expression were reported to be tumor initiators or promoters [28], the malignant potential of CG should be examined in future studies.

In conclusion, COX-2 and Bcl-2 overexpression in CG suggests that CG, particularly the intestinal type, may be a premalignant lesion that converts into a tumor in the presence of carcinogens. However, further molecular and clinical studies are needed to test this hypothesis.

## Conclusion

COX-2 and Bcl-2 overexpression in CG suggests that CG, particularly the intestinal type, may be a premalignant lesion that converts into a tumor in the presence of carcinogens.

## Competing interests

The authors declare that they have no competing interests.

## Authors’ contributions

ZL, GG, and RF performed the molecular genetic studies, analyzed the sequence alignment, and drafted the manuscript. DW performed the immunoassays. BS helped with sequence alignment. XW designed the study and performed the statistical analyses. YC and JL conceived the study, designed the study, and coordinated and drafted the manuscript. XJ conceived the study and designed, coordinated, and drafted the manuscript. All authors read and approved the final manuscript.

## Pre-publication history

The pre-publication history for this paper can be accessed here:

http://www.biomedcentral.com/1471-2490/14/2/prepub
